# *Leptospira* spp. in Domestic Cats from Different Environments: Prevalence of Antibodies and Risk Factors Associated with the Seropositivity

**DOI:** 10.3390/ani4040612

**Published:** 2014-09-29

**Authors:** Lucía Azócar-Aedo, Gustavo Monti, Ronald Jara

**Affiliations:** 1 Graduate School, Faculty of Veterinary Sciences, Universidad Austral de Chile, Valdivia, P.O. Box 567, Chile; E-Mail: luciaazocaraedo@gmail.com; 2 Preventive Veterinary Medicine Department, Faculty of Veterinary Sciences, Universidad Austral de Chile, Valdivia, P.O. Box 567, Chile; 3 Biochemistry and Microbiology Department, Faculty of Sciences, Universidad Austral de Chile, Valdivia, P.O. Box 567, Chile; E-Mail: ronaldjara@gmail.com

**Keywords:** *Leptospira* spp., anti-*Leptospira* antibodies, cats, urban and rural environments, microscopic agglutination test, prevalence, risk factors

## Abstract

**Simple Summary:**

Although *Leptospira* infection occurs in domestic cat populations, studies on leptospirosis are very limited in felines and the role of cats in the epidemiology of this zoonosis has not received much attention. The present work is an epidemiologic study intended to determine the prevalence of anti-*Leptospira* antibodies and risk factors related with the seropositivity in cats from urban and rural environments. A higher prevalence in rural cats was detected (25.2%) compared with urban animals (1.8%). Characteristics of the habitat of the animals and some agricultural activities performed by cat’s owners were found to be risk factors associated with the seropositivity.

**Abstract:**

Leptospirosis is an emerging zoonotic disease of worldwide distribution. A cross-sectional study was conducted in urban and rural environments in southern Chile (1) to detect domestic cats with serologic evidence of exposure to *Leptospira* spp.; (2) to determine the prevalence of anti-*Leptospira* antibodies; (3) to describe seroprevalences according to different characteristics of the animals, and (4) to identify risk factors associated with the seropositivity in the Microscopic Agglutination Test (MAT). Blood samples were taken from 124 owned cats. A frequentist and Bayesian approach were applied for prevalence estimation. The overall apparent prevalence of anti-*Leptospira* antibodies was 8.1% (95% Confident Interval = 3.9–4.3). With the Bayesian approach, the overall True Prevalence (TP) was 5.2% (95% Credibility Interval (CrI) = 0.6–12.4). The TP for urban cats was 1.8% (95% CrI = 0.1–7.2) and the TP for rural felines was 25.2% (95% CrI = 9.3–46.6). Cats that live in a place where agricultural activities are performed with water that flows in streams or backwater and cats that live in places near flooded areas had a higher risk of seropositivity in MAT. The exposure to *Leptospira* spp. in domestic cats of urban and rural origin in Southern Chile is a public health concern that requires an increased awareness and the implementation of preventive measures.

## 1. Introduction

Leptospirosis is an emerging zoonotic disease of worldwide distribution that is caused by spirochaetes of the genus *Leptospira* [1]. Formerly, it was thought that domestic cats were resistant to infections caused by spirochaetes and many practitioners do not consider the feline leptospirosis in the differential diagnosis of other diseases [2]. However, the presence of antibodies have demonstrated that cats can be infected [3] and that they can be incidental hosts of some *Leptospira* serovars that are prevalent in wildlife or in other domestic animals [3], such as Ballum [4], Copenhageni, Hardjo, Icterohaemorragiae [5,6], Rachmati, Bratislava, Bataviae [7], Canicola [4], Autumnalis, and Grippotyphosa [4,6,8].

Due to the limited ability to diagnose *Leptospira* infection in endemic regions worldwide [9], and given that the clinical leptospirosis is difficult to recognize or is less frequent in cats than in other animal species [6], it is possible that the infection may be subdiagnosed in feline populations, for example, in cats that have a history of living outdoor or that have the habit of hunting and the potential risk factors associated with the seropositivity in diagnostic tests for leptospirosis have not been widely investigated in observational studies [3]. Moreover, the role of cats in the epidemiology of this disease has not received much attention [8].

Reliable and updated estimations of the seroprevalence in cats at the national or regional levels are scarce in South America in general [10,11] and in Chile in particular. Only one study [12] has established that cats exposed to *Leptospira* are present in the country. To investigate whether certain characteristics of the cats, their lifestyle or features of their habitat could influence the seropositivity to *Leptospira* spp., the aims of this study were the following: (1) to detect the presence of domestic cats with serologic evidence of exposure to *Leptospira* spp. in urban and rural environments; (2) to determine the prevalence of anti-*Leptospira* antibodies in both environments; (3) to describe prevalences according to the urban and rural origin and the different characteristics of the cats; and (4) to identify risk factors associated with the seropositivity to Microscopic Agglutination Test (MAT).

## 2. Experimental Section

### 2.1. Study Area

The area under study is located between 36°00' and 44°04' south and between 71°00' west and the Pacific Ocean [13]. This region has an area of 48,585 km^2^ and its population, according to the Chilean 2002 Census, was 1,243,000, with a population density of 25.6/km^2^. The region, in general, contains natural vegetation that can be classified as “Valdivian temperate rain forest”. The coastal region, except for the southern portion of Chiloé Island, has a temperate climate with cold winter rain. To the south, the climate is characterized by constant rain and no dry seasons.

Four distinct landscape types or morphological units can be distinguished in the region. These are, from west to east, the Coastal Range, the Intermediate Depression, the Precordillera, and the Andes. These units are oriented parallel to the coast and its subduction zone. 

### 2.2. Study Design and Population Surveyed

Between January 2011 and September 2012, a cross-sectional study was performed using 124 serum samples collected from male and female domestic cats of different breeds that were older than two months of age. Ninety-six of them were from some of the main cities of the Los Rios and the Los Lagos regions in Southern Chile, such as Valdivia and Osorno, but some were from smaller cities, such as Paillaco and San Pablo. The cats were recruited from veterinary clinics, and they were enrolled in the study during home visits or in a veterinary neutering campaign. The samples included animals attending veterinary clinics for various reasons and healthy cats. In addition, 60 dairy farms in the Los Rios and the Los Lagos regions were randomly chosen and 28 cats were sampled from those that were available. There were no previous records about the sizes of the feline populations in those areas for the estimation of a probabilistic sample size and there was no sampling frame for selecting the cats. During the visits to the farms, all cats were sampled.

**Figure 1:**
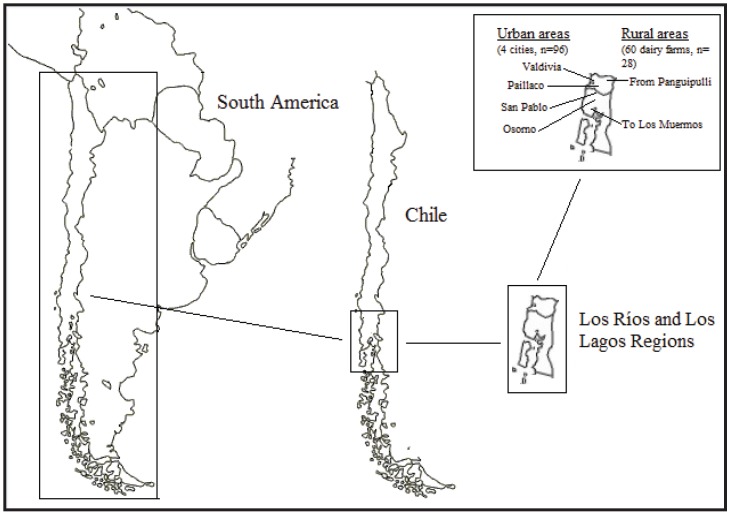
Approximate localization of the Los Rios and the Los Lagos regions in Chile and South America and the study area.

Figure 1 shows the approximate localization of the Los Rios and the Los Lagos regions in Chile and South America and the study area.

### 2.3. Field and Laboratory Procedures

The blood samples were collected by venipuncture (0.5–1 mL). The owners and practitioners voluntarily agreed to participate in the survey.

The owners were interviewed to obtain information about the cats’ individual characteristics (breed, gender, age), lifestyle (purpose of keeping the cats, veterinary control, indoor or outdoor habitat at home, rodent hunting habits, contact with other animals), and habitat (rodents roaming near houses, excreta disposal site of the owners, agricultural activities involving water that flows in streams or backwater by the owners, habitat close to flooded areas). All felines were subjected to a physical examination. An animal was considered as suspected of suffering leptospirosis if they had one or more of the following symptoms: depression, anorexia, fever, icterus and/or renal and/or hepatic alterations in the biochemical profile (when it was available), such as increased serum urea or creatinine, alanine aminotransferase, aspartate aminotransferase, and/or alkaline phosphatase.

The exposure to *Leptospira* spp. was detected using MAT. The 124 samples were analyzed at the Bacteriology Laboratory located in the Biochemistry and Microbiology Department of the Faculty of Sciences at the Universidad Austral de Chile in Valdivia-Chile following standard guidelines [14]. The MAT was performed using live cultures of *L. interrrogans* serovars Hardjo, Pomona, Canicola, Icterohaemorragiae, and Autumnalis and *L. borgpetersenii* serovar Ballum. These serovars were included in the panel of antigens because they are among those that occur in animals in Chile according to bibliographic data [15] and the experience of the laboratory.

To broaden the panel of the serovars, 40 of the MAT-negative samples were re-analyzed using 12 different serovars at the National Institute of Respiratory Diseases “Dr. Emilio Coni” in Santa Fe-Argentina using a methodology previously proposed [14,16]. The panel included the following serovars: *L. interrogans* serovars Pyrogenes, Wolffi, Bataviae, Australis, and Hebdomadis; *L. borgpetersenii* serovars Tarassovi, Javanica, and Sejroe; *L. kirschneri* serovars Grippotyphosa and Cynopteri; *L. noguchii* serovar Panama; and *L. biflexa* serovar Patoc. The reference strains for each serovars and serogroups are listed in Table 1.

A titre of 1:100 or higher was considered as indicative of exposure to *Leptospira* spp. For cats that reacted to MAT for more than one serovar, the serovar associated with the highest titre was specified as the cause of the seropositivity and reactions to different serovars at the same titre were considered coagglutinations.

### 2.4. Ethical Considerations

The methods for animal handling and blood extraction were used in the first author doctoral thesis, which followed the guidelines for animal management and welfare and was approved by the bioethics committee at the Universidad Austral de Chile, by obtaining the “Certificate for Use of Animals in Research” (certification number #10-2012).

**Table 1:** Serogroups, serovars and reference strains of *Leptospira* species used for Microscopic Agglutination Test (MAT).

Species	Serogroups	Serovar	Reference strain
Panel 1 *:			
*L. interrogans*	Sejroe	Hardjo	Hardjo pratijno
	Pomona	Pomona	Pomona
	Canicola	Canicola	Hond Utrech IV
	Icterohaemorragiae	Icterohaemorragiae	Verdun
	Autumnalis	Autumnalis	Akiyami A
*L. borgpetersenii*	Ballum	Ballum	S102
Panel 2 **:			
*L. interrogans*	Pyrogenes	Pyrogenes	Salinem
	Sejroe	Wolfii	3705
	Batavie	Bataviae	Swart
	Australis	Australis	Ballico
	Hebdomadis	Hebdomadis	Hebdomadis
*L. borgpetersenii*	Tarassovi	Tarassovi	Perepelitsin
	Javanica	Javanica	Veldrat Batavia 46
	Sejroe	Sejroe	M 84
*L. kirschneri*	Grippotyphosa	Grippotyphosa	Moskva V
	Cynopteri	Cynopteri	3522 C
*L. noguchii*	Panama	Panama	CZ 214K
*L. biflexa*	Semaranga	Patoc	Patoc 1

* 124 samples were analyzed with this panel in Chile, ** 40 samples were analyzed with this panel in Argentina.

### 2.5. Data Analysis

The Apparent Prevalence (AP) of anti-*Leptospira* antibodies for all cats as a group and for cats of urban and rural environments separately, as well as the prevalence according to cat’s individual, lifestyle, and habitat characteristics were estimated based on MAT results using the methodology proposed by Fletcher and Fletcher [17].

### 2.6. Prevalence of Anti-Leptospira Antibodies, Taking into Account the Diagnostic Error

A Bayesian approach was used to estimate the True Prevalence (TP) in the population, assuming that the AP provided information based on an imperfect diagnostic test, leading to the possibility of both false positive and false negative ascertainment of *Leptospira* exposure status. Therefore, the uncertainty about the sensitivity (Se) and specificity (Sp) of the diagnostic test was modeled using independent beta prior distributions, as follows: *Se ~ Beta(a_Se_,b_Se_) Sp ~ Beta(a_Sp_,b_Sp_)* in which the prior parameters of the *beta* distributions were estimated based on the most likely (modal) prior value of the parameter and an upper or lower percentile for the parameter [18]. Because there was no information available for the test used for cats, the prior values were based on a review of the international literature. For the different tests, the Se and Sp parameters were modeled using a mode and assuming that we were 95% certain that each was greater than a certain value. 

An “optimistic” prior distribution was set for a very high Sp value of 96.4% and a Se value of 98.2% [19,20] (Model 1). To evaluate the robustness of our estimates, two additional Se priors were used: a “skeptical” prior for Sp and Se of 49.9% (Model 2) and a combination of an “optimistic” Sp of 95% with a “skeptical” prior for Se as suggested by Limmathurotsakul *et al.* [20] (Model 3). In addition, different priors (Uniform and BetaPert) were used to assess the robustness of the results for the choice of a prior. The priors used are performance measures for MAT reported in two studies carried out in humans. It were selected because to date there are no estimations of the diagnostic Se and Sp for MAT in cats or other animal species.

The convergence value of the models was assessed using the Brooks-Gelman-Rubin (BGR) statistic. The estimations were performed using the R package Prevalence [21], and 200,000 iterations were run after a burn-in of 10,000 iterations, which were discarded. The median of the posterior distribution and the 95% Credibility Intervals (CrI) of the parameters of interest were estimated.

### 2.7. Statistical Analysis

The differences in the characteristics of MAT-reactive animals (seropositive and seronegative cats) according to the urban or rural origin were analyzed by the chi-square test for statistical significance (*p* < 0.05) with EpiInfo version 6.04.

The assessment of the association between potential risk factors, such as the different individual, lifestyle and habitat characteristics of the cats and the seropositivity in MAT were performed using conditional logistic regression models. Three models were built, one for all of the animals and one for either the cats of urban origin or those of rural origin.

The variables were first selected using unconditional logistic regression with each variable (*p* < 0.25) and then conditional models were constructed using a forward strategy for variable inclusion and the Akaike’s Information Criteria (AIC) were used for assessing the goodness-of-fit of the different models. Odds Ratios (OR) and their 95% confidence intervals (CI) for the variables included in the final model were estimated and a *p*-value of <0.05 was used for assigning statistical significance. Additionally, interactions between the variables were evaluated on the basis of biological plausibility, as well as potential confounders. The predictive ability and performance of the models were evaluated using the area under the Receiver Operating Characteristics (ROC) curve and the D_xy_ Sommer’s Rank Correlation (D_xy_ indicator) values. All analyses were performed using R version 2.15.1.

## 3. Results and Discussion

### 3.1. Prevalence of Anti-Leptospira Antibodies in Different Cat Populations

To date, there have been few prevalence studies considering serologic evidence of exposure to *Leptospira* spp. in cats worldwide and, in particular, in South America and Chile. The present study provides the first estimate of the true prevalence of antibodies in felines in Chile.

From a total of 124 cats sampled, 10 were found with anti-*Leptospira* antibodies using MAT. Therefore, the AP of anti-*Leptospira* antibodies was 8.1% (95% CI = 3.9–14.3). Most of the cats that were serologically reactors in the diagnostic test were from rural areas and none had clinical signs of leptospirosis (Table 2). The AP of anti-*Leptospira* antibodies in urban areas was 3.1% (95% CI = 0.6–8.9) and in rural areas, the AP was 25.0% (95% CI = 10.7–44.9). Consistent prevalence estimations were observed in epidemiological surveys in England (6.8%) [22], Japan (7.7%) [23], and Scotland (9.2%) [6], but lower seroprevalences has been reported in the US (4.8%) [8] and Iran (4.9%) [4,24]. A higher rate was observed in Greece (33.3%) [7,25], but is important to consider that most of epidemiologic studies to date have reported prevalences, without specifying the urban or rural origin of the animals. 

**Table 2:** Summary of the individual characteristics, origin, serovars, and antibody titres of 10 positive cats tested using MAT.

Cat No.	Breed *	Gender **	Age (Years)	Clinical signs	Origin	Antibody Titre	Serovar
1	DSH	M	1	No	Urban	1:100	Canicola
2	DSH	F	4	No	Urban	1:100	Autumnalis
3	DSH	M	4	No	Urban	1:200	Coagglutination 1 ***
4	DSH	F	1	No	Rural	1:100	Canicola
5	DSH	F	6	No	Rural	1:100	Autumnalis
6	DSH	F	3	No	Rural	1:100	Autumnalis
7	DSH	F	5	No	Rural	1:100	Grippotyphosa
8	DSH	F	0.5	No	Rural	1:200	Bataviae
9	DLH	M	3	No	Rural	1:400	Bataviae
10	DSH	F	2	No	Rural	1:100	Coagglutination 2 ***

* DSH: Domestic Short Hair breed cat, DLH: Domestic Long Hair breed cat, ** F: Female, M: Male, *** Coagglutination 1: serovars Wolffi/Bataviae, *** Coagglutination 2: serovars Grippotyphosa/Wolffi/Bataviae/Sejroe/Javanica/Panama.

Studies from Brazil provided a similar estimated seroprevalence of 11.0% [10] and a higher seroprevalence of 22.3% [11] compared with the present study, but the latter study used only a small number of cats from a municipal shelter for stray animals. In Chile, a survey was performed in the city of Chillán (in the south-central region of Chile) using a sample of 20 animals from a veterinary hospital. It was reported that four of these cats (20.0%) were serological reactors to *Leptospira* and two of them were evaluated using MAT and only one was positive, with a titre of 1:40 for serovar Canicola [12]. Although it has been suggested that large variations in the *Leptospira* seroprevalence among different areas of a country are possible [26], given the small sample size and the way the authors obtained the cats, it is not possible to compare the findings of the study in Chillán with the results of the present study. 

Specific estimations of MAT diagnostic performance for cats are lacking; this information is important to determine. This knowledge will allow adjustments for misclassifications, which is essential for obtaining accurate prevalence estimates and is needed for planning future health service delivery to individuals with cats and their families. With accurate and reliable data, better control strategies could be designed and applied. However, using a Bayesian approach makes it possible to intuitively take into account some of the uncertainty in the data and the diagnostic test used. This methodology for the estimation of the TP has been used in prevalence studies on a number of different conditions [27,28], but it has not been applied frequently to *Leptospira* infection. Table 3 shows the estimates of the TP (overall, for urban and rural cats) and the 95% CrI that were obtained. In the three models, the prevalence point estimates were higher in rural cats compared with the TP in all cats and in urban animals, although the credible intervals were wide and overlapping. All of the BGR values were near 1 or not substantially greater than 1. The values of the model 1 (“optimistic”) were considered as the best estimate of the TP considering that MAT is the reference test for the serologic diagnosis of leptospirosis and its diagnostic performance is expected to be close to these values.

**Table 3:** Estimates of the True Prevalence (TP) (overall, urban and rural cats) and their 95% Credibility Intervals (CrI) that were obtained using a Bayesian approach, considering different models of the uncertainty for MAT-based sensitivity (Se) and specificity (Sp) values.

TP (%)	Model 1	Model 2	Model 3
Overall	5.2 (0.6; 12.4)	2.2 (0.1; 9.0)	4.0 (0.2; 16.8)
Urban	1.8 (0.1; 7.2)	1.3 (0.05; 6.2)	2.4 (0.1; 11.6)
Rural	25.2 (9.3; 46.6)	8.5 (0.3; 33.1)	26.3 (1.5; 82.0)

The prevalence of anti-*Leptospira* antibodies can vary according to several factors, such as the following: the *Leptospira* serovar causing the serological reaction in the diagnostic test, because few serovars are endemic to a particular geographic location [1,29]; the age of the animals, considering that young individuals could be more vulnerable to the disease [26,29]; the population density, because the infection can be related to overcrowding, poor hygienic standards and inadequate sanitation [30], and contact with rodents or other wildlife carriers of leptospires [5,31]. Additionally, the time of the year (season) when the sampling was conducted could explain the differences between the results of different studies, given that leptospirosis is prevalent in geographic regions with high annual rainfall and a warm climate. In conclusion, differences in environmental conditions, which allow different patterns of transmission and dynamics of *Leptospira* infection in the feline population, different study designs, the characteristics of the animals included in the surveys and the diagnostic tests used can affect the results of the studies.

The AP of anti-*Leptospira* antibodies in cats of rural origin was 25.0%, in contrast with the low prevalence observed in urban cats (3.1%). In rural settings, a high risk of seropositivity could be expected because these environments often contain livestock, rodents and small mammals, which are usual reservoirs of leptospires [1]. Indirect contact between leptospires and susceptible animals could occur from soil and water contaminated with the urine of *Leptospira* carriers. Thus, the high seroprevalence observed in rural areas is not surprising and the finding of MAT serological reactors in the urban areas indicated the spread of the exposure to the bacterium into different environments.

### 3.2. Serovars, Antibody Titres and Prevalences According to Different Characteristics of the Cats

The knowledge about the prevalent serovars and their maintenance hosts is important for understanding the epidemiology of leptospirosis in a particular geographic area [31]. In this study, the cats were reactive to *L. interrogans* serovars Autumnalis (serogroup Autumnalis) (3/10, 30.0%), Canicola (serogroup Canicola) (2/10, 20.0%) and Bataviae (serogroup Bataviae) (2/10, 20.0%) and to *L. kirschneri* serovar Grippotyphosa (serogroup Grippotyphosa) (1/10, 10.0%). There was a coagglutination with two serovars (10.0%) and one with six serovars (10.0%) (Table 2). However, the rural and urban cats did not react to the same serovars. All of the urban and some of the rural cats shared exposure to *L. interrogans* serovars Autumnalis and Canicola, but *L. interrogans* serovar Bataviae and *L. kirschneri* serovar Grippotyphosa were found only in rural cats.

Table 4 shows the prevalence of anti-*Leptospira* antibodies according to individual, lifestyle and habitat characteristics for all cats and for urban or rural animals. Statistically significant differences between MAT-seropositive and seronegative animals were only found for cats in contact with livestock (*p* < 0.05).

**Table 4:** Prevalence of anti-*Leptospira* antibodies based on MAT results according to individual, lifestyle and habitat characteristics of cats from different environments.

Characteristics	*Categories*	All	Urban	Rural
		(%)	(%)	(%)
Breed	*DSH*	9.3 (9/97)	4.1 (3/73)	25.0 (6/24)
	*DLH*	4.3 (1/23)	0.0 (0/20)	33.3 (1/3)
	*Other breeds*	0.0 (0/4)	0.0 (0/3)	0.0 (0/1)
Gender	*Male*	5.5 (3/55)	4.7 (2/43)	8.3 (1/12)
	*Females*	10.1 (7/69)	1.9 (1/53)	37.5 (6/16)
Age	*<1 year*	5.0 (2/40)	0.0 (0/31)	22.2 (2/9)
	*1 to 3 years*	9.8 (5/51)	2.9 (1/35)	25.0 (4/16)
	*3 to 6 years*	12.5 (3/24)	9.5 (2/21)	33.3 (1/3)
	*6 to 9 years*	0.0 (0/7)	0.0 (0/7)	0.0 (0/0)
	*>10 years*	0.0 (0/2)	0.0 (0/2)	0.0 (0/0)
Purpose of kept cats	*Companion*	4.3 (3/70)	4.4 (3/68)	0.0 (0/2)
	*Rodents hunting*	6.3 (1/16)	0.0 (0/8)	12.5 (1/8)
	*Companion/hunting*	16.7 (6/36)	0.0 (0/18)	33.3 (6/18)
Veterinary control	*Yes*	3.4 (2/59)	0.0 (0/50)	22.2 (2/9)
	*No*	12.3 (8/65)	6.5 (3/46)	26.3 (5/19)
Habitat at home	*Indoor/outdoor*	4.9 (4/81)	4.1 (3/74)	14.3 (1/7)
	*Indoor*	0.0 (0/9)	0.0 (0/9)	0.0 (0/0)
	*Outdoor*	17.6 (6/34)	0.0 (0/13)	28.6 (6/21)
Rodent-hunting habits	*Yes*	12.1 (7/58)	0.0 (0/33)	28.0 (7/25)
	*No*	3.2 (2/63)	3.3 (2/60)	0.0 (0/3)
Contact with other cats	*Yes*	5.3 (5/95)	1.3 (1/77)	22.2 (4/18)
	*No*	13.0 (3/23)	7.1 (1/14)	22.2 (2/9)
Contact with livestock (cattle, sheep and/or goats)	*Yes*	28.6 (6/21)	0.0 (0/1)	30.0 (6/20)
	*No*	3.1 (3/98)	2.2 (2/90)	12.5 (1/8)
Presence of dogs at home	*Yes*	7.3 (7/96)	1.3 (1/75)	25.0 (7/28)
	*No*	7.7 (1/13)	7.1 (1/13)	0.0 (0/0)
Rodents roaming near houses	*Yes*	9.6 (5/52)	2.6 (1/39)	30.8 (4/13)
	*No*	7.4 (5/68)	3.8 (2/53)	20.0 (3/15)
Excreta disposal site of the owners	*Sewers*	6.1 (7/114)	3.2 (3/93)	19.0 (4/21)
	*Outhouses*	30.0 (3/10)	0.0 (0/3)	42.9 (3/7)
Activities with water that flows in streams or backwater	*Yes*	33.3 (2/6)	0.0 (0/0)	33.3 (2/6)
	*No*	6.8 (8/118)	3.1 (3/96)	22.7 (5/22)
Habitat close to flooded areas	*Yes*	40.0 (2/5)	0.0 (0/0)	40.0 (2/5)
	*No*	7.1 (8/113)	3.3 (3/90)	21.7 (5/23)

Because most of the serological reactive cats to MAT were found among those living outdoor (6/34), with hunting habits (7/58) and with the presence of rodents roaming near houses (5/52) (Table 4), it could explain their exposure to serovar Autumnalis. The known primary reservoir for the serovar Autumnalis is mice [32]. A prey-predator chain of leptospiral infection was demonstrated in mice and cats in New Zealand for serovar Ballum [5] and this chain is likely to exist for serovar Autumnalis as well and infections with this serovar were also diagnosed in cats in the US [8] and Brazil [10].

The maintenance hosts for serovar Canicola are mainly dogs and the primary hosts for serovar Bataviae are dogs, rats, and mice. Dogs are considered important reservoirs for pathogenic serovars because these animals develop leptospiuria of high intensity and long duration [32]. Most of the reactive cats in the MAT in this study lived with dogs (7/96) and had rodent-hunting habits (7/58), consequently, the contact with dogs, rats and/or mice could be the most likely source of the exposure to serovars Canicola and Bataviae. Infections with the serovar Canicola were also detected in felines in Iran [4,24] and Greece [7], and infections with the serovar Bataviae were found in cats in Brazil [11].

The antibody titres in serological reactive cats ranged from 1:100 to 1:400. Seven cats had an antibody titre of 1:100, 2 had a titre of 1:200 and 1 had a titre of 1:400 (Table 2). Because it was not possible to obtain a second serum sample to determine an increase in the antibody titres, or urine for bacterial detection, we cannot confirm that felines are indeed infected and shed the bacterium or whether they may respond to an infection with low antibody levels that decrease with time, as mentioned by Jamshidi *et al.* [5], but in general, the antibody titres of cats in others studies have also been low [4,6,11].

All cats exposed to *L. interrogans* serovars Autumnalis and Canicola and *L. kirschneri* serovar Grippotyphosa exhibited antibody titres of 1:100; however, two rural cats exposed to *L. interrogans* serovar Bataviae showed higher antibody titres (1:200 and 1:400) (Table 2). Titres of 1:200 and higher could be related to a clinical disease; however, none of the seropositive cats had clinical signs of leptospirosis. Under experimental conditions, *Leptospira* infection could not induce symptoms in domestic felines [33,34]; therefore, positive test results in this species must be interpreted as indicative of exposure with leptospires rather than as disease.

A higher AP was observed in cats with rodent hunting habits, particularly among the rural felines (7/25). Cats are known to be skilled hunters of mice and rats, which potentially carry *L. interrogans*, allowing opportunities to become infected [5]. Interestingly, cat ownership was associated with a lower risk of human leptospirosis in one study and the authors concluded that the presence of cats could reduce the risk of rodent-borne leptospirosis [35].

### 3.3. Risk Factors Associated with the Seropositivity in MAT

The variables included in the conditional logistic regression analysis that considered all animals are listed in Table 5 (model 1). Those statistically significant were: (1) being a cat that lives in places when the owners perform agricultural activities with water that flows in streams or backwater, and (2) being a cat that lives near flooded areas.

**Table 5:** Conditional logistic regression models to identify risk factors associated with the seropositivity in MAT.

Variables Included in the Models	*Categories*	OR	95% CI
Model 1: all cats ***			
Contact with cattle, sheep and/or goats:	*Yes*	1.9	0.1–139.8
	*No*	Ref *	
Contact with other cats:	*Yes*	0.1	0.1–1.7
	*No*	Ref *	
Excreta disposal site of the owners:	*Outhouse*	5.5	0.2–166.6
	*Sewers*	Ref *	
Activities with water that flows in streams or backwater:	*Yes*	38.0	1.9–763.9 **
	*No*	Ref *	
Habitat near flooded areas:	*Yes*	44.5	1.4–1450.5 **
	*No*	Ref*	
Veterinary control:	*Yes*	0.1	0.1–2.4
	*No*	Ref *	
Rural origin:	*Yes*	1.4	0.1–241.9
	*No*	Ref *	
**Model 2: urban cats** ***			
Contact with other cats:	*Yes*	0.12	0.1–2.2
	*No*	Ref *	
**Model 3: rural cats** ***			
Gender:	*Male*	0.1	0.1–1.3
	*Female*	Ref *	
Habitat indoor and outdoor:	*Yes*	0.1	0.0–1.9
	*No*	Ref *	

*** The area under the ROC Curve and the D_xy_ value were 0.893 and 0.787, respectively for model 1 (all cats), 0.694 and 0.388, respectively, for model 2 (urban cats) and 0.772 and 0.544, respectively, for model 3 (rural cats), ** Statistically significant (*p* < 0.05), * Reference category.

Cats that dwelled in places where their owners performed agricultural activities using water from streams or backwater were 38 times more likely to be seropositives in MAT (Table 5). Eco-friendly conditions allow the maintenance of leptospires in the environment. Water is considered an important environmental factor in the maintenance of leptospires and once contaminated with urine from infected animals, it is a significant factor in the transmission of the infection [1,36,37]. The leptospires can survive in the environment for long periods, particularly under warm and humid conditions [1]. If water is extracted from natural aquatic sources for irrigation of gardens or orchards, for example, or for other agricultural activities, it is possible that this water will be contaminated and that the bacteria will survive in the humid soil. Considering that most of the positive felines in this study dwelled in outdoor areas (6/34), their contact with irrigated areas could explain their higher risk of seropositivity in the diagnostic test.

Deficiencies in basic sanitation, inadequate waste management and the presence of stagnant water near homes affect the possible transmission of leptospires in the environment [38]. Among all cats, the animals that lived in a place close to flooded areas have 44.5 more probabilities of seropositivity in MAT (Table 5). Outbreaks of leptospirosis in humans frequently have occurred in endemic areas in those who have been exposed to floodwaters, which are very likely to have leptospiral contamination [38]. Domestic dogs located in an area with frequent flooding were associated with leptospirosis in Brazil [10] and the U.S. [36,37]. 

The conditional logistic regression models for urban and rural cats by separately are showed in Table 5 (models 2 and 3). Risk factors statistically associated with the seropositivity in MAT were not found in these models. Nevertheless, in the urban cats, the variables that were included in the unconditional model were contact with other felines (OR = 0.1; 95% CI = 0.1–2.2) and the presence of dogs at home (OR = 0.2; 95% CI = 0.1–2.7). In the rural cats, these variables were being a male cat (OR = 0.1; 95% CI = 0.1–1.5), being a non-neutered cat (OR = 0.1; 95% CI = 0.1–1.7), living indoor/outdoor (OR = 0.2; 95% CI = 0.2–2.2), living outdoor (OR = 4.5; 95% CI = 0.5–44.3), coming into contact with livestock (OR = 4.5; 95% CI = 0.5–44.3), and being a cat that lives in a place where the excreta disposal sites of the owners were sewers (OR = 4.5; 95% CI = 0.6–31.1). None of these variables were statistically significant in the conditional analysis. This study may not have had sufficient power to detect differences, which could be due to the small sample size and the low number seropositive felines in the diagnostic test or because other than the studied variables could be related to the infection, therefore further studies are needed to clarify the risk factors associated with the MAT seropositivity in MAT in felines from urban and rural environments.

In this study since MAT was performed with single serum samples, we cannot define if the presence of anti-*Leptospira* antibodies corresponds to past or recent infections, but the finding of seropositive cats without clinical evidence of disease arise the question regarding the potential risk of zoonotic transmission of the bacterium, considering that in a recent study [39] the urinary shedding of leptospires was confirmed in healthy felines and in animals with renal disease and in other survey [34], leptospiuria for at least two to eight weeks was detected in serological reactive cats to *Leptospira.* Therefore, is important to determine the role of felines exposed to *Leptospira* as a source of infection and environmental contamination with the bacterium with bacteriological or molecular diagnostic tests of urine.

## 4. Conclusions

Domestic cats from urban and rural environments in southern Chile are exposed to leptospires and they might play a role in the epidemiology of the disease. The proportion of serological reactors in the diagnostic test was more frequent among cats of rural origin and in animals that had certain characteristics and environmental conditions in their habitats. Knowing the characteristics of cats that are associated with the exposure to *Leptospira* could be useful for identifying suspected patients in feline clinical practice.

These results also highlight the public health concern that arises from the presence of anti-*Leptospira* antibodies in cats in urban and rural areas, making it necessary to increase the awareness of *Leptospira* infection among veterinarians and pet owners and to instigate appropriate preventive measures in cats with a higher risk of infection and in other feline populations.
